# Autoimmune Polyendocrine Syndromes in Adult Italian Celiac Disease Patients

**DOI:** 10.3390/jcm13020488

**Published:** 2024-01-16

**Authors:** Dante Pio Pallotta, Alessandro Granito, Alberto Raiteri, Maria Boe, Agnese Pratelli, Alice Giamperoli, Giovanni Monaco, Chiara Faggiano, Francesco Tovoli

**Affiliations:** 1 Unit of Internal Medicine, Hepatobiliary and Immunoallergic Diseases, IRCCS Azienda Ospedaliero-Universitaria di Bologna, 40138 Bologna, Italy; dantepio.pallotta@studio.unibo.it (D.P.P.); alberto.raiteri@studio.unibo.it (A.R.); maria.boe@studio.unibo.it (M.B.); agnese.pratelli@studio.unibo.it (A.P.); alice.giamperoli@studio.unibo.it (A.G.); giovanni.monaco3@studio.unibo.it (G.M.); chiara.faggiano@aosp.bo.it (C.F.); francesco.tovoli@unibo.it (F.T.); 2 Department of Medical and Surgical Sciences, University of Bologna, 40138 Bologna, Italy

**Keywords:** celiac disease, autoimmune endocrinopathy, autoimmune polyendocrine syndrome, extraintestinal autoimmune diseases, thyroiditis, type-1 diabetes mellitus, Addison’s disease, autoantibodies

## Abstract

Celiac disease (CD) is frequently associated with other autoimmune disorders. Different studies have explored the association between CD and single autoimmune endocrine disease (AED), especially autoimmune thyroiditis (AIT) and type-1 diabetes mellitus (T1DM). Data about CD as a component of autoimmune polyendocrine syndrome (APS) are scant. We analyzed a large dataset including prospectively collected data from 920 consecutive adult CD patients diagnosed in a third-level Italian institution in the 2013–2023 period, The prevalence of isolated autoimmune endocrine diseases and APS were collected. A total of 262 (28.5%) CD patients had at least one associated AED, with AIT (n = 223, 24.2%) and T1DM (n = 27, 2.9%) being the most frequent conditions. In most cases (n = 173, 66%), AEDs were diagnosed after CD. Thirteen patients (1.4%) had at least two of the requested three endocrinopathies, satisfying the diagnosis of APS. APS is a rare but not exceptional occurrence among Italian CD patients, underscoring the intricate and multifaceted nature of autoimmune disorders. Periodic evaluations of thyroid function and glycaemia should be recommended after the diagnosis of CD together with testing for autoantibodies that may be helpful in assessing disease risk before disease onset. Likewise, implementation of a systematic screening for CD amongst T1DM and other autoimmune endocrine diseases are paramount.

## 1. Introduction

Celiac disease (CD) is a chronic small intestinal autoimmune enteropathy precipitated by exposure to dietary gluten in genetically predisposed individuals [1]. Patients with CD are at higher risk of developing other autoimmune diseases, including autoimmune endocrinopathies [2]. This increased risk is explained by the tendency of autoimmune disease to cluster and the particular vulnerability of the endocrine system to autoimmune insults [3].

Several studies analyzed the incidence of autoimmune endocrine disorders (AEDs) in patients with CD and the occurrence of CD in patients with AED [4,5,6]. Autoimmune thyroid disorders and type 1 diabetes mellitus (T1DM) are the most common AEDs both in the general and CD populations. Other AEDs, such as Addison’s disease (AD) and autoimmune pituitary disorders, have been associated with CD [2,7]. The co-occurrence of multiple AEDs defines the spectrum of autoimmune polyendocrine syndromes (APS) [8].

These syndromes can be broadly categorized either as rare monogenic forms such as APS type 1 (APS-1) and X-linked immunodysregulation, polyendocrinopathy, and enteropathy (IPEX), or more common polygenic varieties, i.e., APS type 2 (APS-2) [9].

In 1980, Neufeld proposed a clinical classification of APS based on four types, the very rare juvenile type 1 and the more frequent adult types 2 to 4 [10].

Types 2 to 4 are adult forms, with a common manifestation between ages 40 and 60 years. Females are affected more often than males, with an M/F ratio of 1:3. Chronic candidiasis is not present in adult APS.

Type 2 APS is characterized by the presence of AD with at least one other autoimmune endocrine disorder that can be either autoimmune thyroid disease or T1DM, or both. Further endocrine (hypoparathyroidism) and nonendocrine (autoimmune gastritis, celiac disease, rheumatoid arthritis, vitiligo, psoriasis, urticaria, alopecia) component diseases can be present. Type 3 APS is defined by the presence of autoimmune thyroid disease and T1DM without affecting the adrenal gland. Type 4 APS is a diagnosis of exclusion, with two or more organ-specific autoimmune endocrine syndromes that cannot be assigned to APS2 or APS3 [10,11].

Importantly, CD and other non-endocrine autoimmune conditions (such as autoimmune gastritis, primary biliary cholangitis, vitiligo, Sjögren syndrome, myasthenia gravis, alopecia, and pernicious anemia) can be additional manifestations in patients with APS3 and APS4 [11].

However, data about the association and relationship between CD and APS are scant. Therefore, we investigated the prevalence of isolated AED and APS amongst a large cohort of Italian adult CD patients.

## 2. Materials and Methods

### 2.1. Patients

For the purposes of this study, we considered all clinical, laboratory, histological and genetic data, prospectively collected during a 10-year period (January 2013–June 2023), at the tertiary referral Celiac Disease Center of the IRCCS S. Orsola Malpighi Polyclinic, University of Bologna (Bologna, Italy).

All patients underwent serological testing and duodenal biopsy within 6 weeks of each other.

The diagnosis of CD was based on serologic testing of celiac-specific antibodies (anti- transglutaminase IgA, or IgG in patients with IgA deficiency) and confirmed by duodenal multiple mucosal biopsies (at least four well-oriented samples, two taken from the duodenal bulb and two from the second portion) [12].

Anti-tissue transglutaminase (tTGA) antibodies have been detected by a standardized and reliable commercial ELISA kit (Eurospital, Trieste, Italy). Antiendomysial (EmA) IgA antibody detection was performed via a commercial indirect immunofluorescence (IFL) assay using monkey esophagus as substrate (Eurospital, Trieste, Italy).

Duodenal biopsies were evaluated by an experienced pathologist and were graded according to Marsh–Oberhüber classification. Intraepithelial lymphocyte (IEL) count was performed using CD3 immunostaining and the villous height/crypt depth ratio was also assessed.

A detailed HLA typing including HLA-DQ2.5 (DQA1*0501, DQB1*0201), HLADQ8 (DQA1*03, DQB1*0302), HLA-DQ 2.2 (DQA1*0201, DQB1*0202) has been performed, when necessary, as recommended [1,13].

Potential CD, the non-atrophic variant of gluten-sensitive enteropathy, was diagnosed in patients with serum endomysial (EmA) and tissue transglutaminase IgA antibodies (tTGA) with positivity for HLA-DQ2 and/or HLA-DQ8 genotype [13].

Data regarding the diagnosis of concurrent autoimmune disease (occurring both before and after the diagnosis of CD) were prospectively collected and reviewed.

Exclusion criteria were the following: (1) incomplete/missing clinical data at diagnosis; (2) no clinical or laboratory follow-up; (3) patients lost to the follow-up. All data were anonymously processed and analyzed.

### 2.2. Statistics

Continuous variables were expressed as median and range, while ordinal and categorial variables were expressed as absolute values and percentages. Statistical analyses were performed using IBM SPSS version 22.0.

### 2.3. Ethics

The protocol of our study was approved by local ethics committee (Protocol 427/2021/Oss/AOUBo, approved by the CE-AVEC ethics committee on 10 January 2021). All patients signed an informed consent prior to their participation to this study. This study was conducted according to the latest Declaration of Helsinki.

## 3. Results

Data from 920 prospectively enrolled adult patients with an established diagnosis of CD were analyzed. Our population included 712 (77.4%) females and 208 (22.6%) males. The median age at the diagnosis of CD was 37.2 years (range 16–84). Associated autoimmune diseases were observed in 299 (32.5%) patients, most of which were AED (n = 262, 28.5%). The most common AEDs were autoimmune thyroiditis (AIT, n = 223, 24.2%) and T1DM/latent autoimmune diabetes of the adult (LADA) (n = 27/2, 3.1%).

Less common observed autoimmune endocrinopathies were Graves’ disease (n = 21, 2.3%), AD (n = 1, 0.1%), and autoimmune hypophysitis (n = 1, 0.1%), as described in Table 1.

Overall, AEDs were more frequently diagnosed after CD diagnosis and gluten-free diet initiation both when AEDs were analyzed tout court (n = 173, 66.0%) and when the diagnoses of AIT (n = 143, 64.1%), T1DM (n = 21, 75.0%), and Graves’ disease (n = 16, 76.6%) were considered separately. Among the 89 (34.0%) patients with pre-existing AED, 54 (60.6%) had later been diagnosed with CD thanks to the case-finding strategy for concurrent classical CD-related symptoms, whereas in 35 (39.4%) of them, CD presented with non-classical clinical manifestations, and thus, the diagnosis was based on the incidental finding of positive CD serology. Most of the latter patients were affected by AIT or T1DM. The demographics of patients with associated autoimmune diseases or AE are reported in Table 2.

Table 4 describes the prevalence of adult APS subtypes based on the combination of AED, as previously described [10,11].

## 4. Discussion

Autoimmune comorbidities are a common occurrence in patients with CD, and the endocrine system represents an especially susceptible target for autoimmunity [14].

In this study, we reported the prevalence of APS and analyzed the clinical features of CD-associated APS found in a large cohort of Italian CD outpatients diagnosed and followed-up to a tertiary referral center. We found a 1.4% prevalence of APS, with the majority of CD patients belonging to the APS type 3, as expected in an adult population [10,11].

Apart from a few cohort studies that have described the prevalence of CD in autoimmune polyglandular syndromes, to the best of our knowledge, no systematic assessments of APS prevalence in Italian CD adult patients have been reported so far [15,16,17].

In a recent retrospective study exploring the frequency of autoimmune diseases in a cohort of 561 APS-2 Italian patients, CD was found in 2% of patients after transglutaminase autoantibodies serological screening. Notably, 77% had developed CD after AD, and in only 23% of cases was the diagnosis of CD made before AD at a mean age of 28 years [18].

In contrast, we found only 1 out of the 13 APS cases with AD, and this could be explained by having studied a CD adult population in whom APS-2 developed subsequently.

In fact, starting from patients diagnosed with CD, we selected a predominantly DQ2-positive and less frequently DQ8-positive study population. DQ2-positive Italian CD patients in about 40% of cases do not possess DRB1*03, which is significantly associated with APS-2 [11,18].

Although we do not have a systematic assessment of our patients’ HLA profile as it is not always required in clinical practice, this different genetic background, due to an inherent characteristic of the enrolled patients, could explain the rarity of AD in our adult CD population.

The incidence of types 2 to 4 ranges between 1.4 and 4.5 per 100,000 depending on the published data source. However, due to the heterogeneous expression pattern, it is presumed that there are a larger number of unreported cases. The actual incidence is thus estimated to be 1:20,000 [10,19].

It is worth mentioning that after Neufeld first attempted to classify APS, in subsequent years, other new classifications of APS were proposed. These new attempts are based on certain considerations including the increase in the number of diseases recognized as autoimmune resulting in more complex combinations of APS and also the proposal to include the association of subclinical and/or potential autoimmune diseases in APS [20].

Furthermore, the initially proposed terms of APS were not appropriate to define these conditions because they could include not only endocrine diseases but also variable combinations of endocrine and non-endocrine autoimmune diseases, or only non-endocrine autoimmune diseases (such as systemic lupus erythematosus and Sjögren’s syndrome, autoimmune gastritis or other autoimmune gastrointestinal, hepatic, or pancreatic diseases and vitiligo).

This led to the proposal that a more appropriate term for these conditions might be multiple autoimmune syndromes (MAS) [21].

In this perspective, celiac disease could constitute a frequent nonendocrine autoimmune condition to be regarded in new classification criteria.

The link between CD and polyendocrine autoimmunity raises questions about the opportunity of screening for AED amongst CD patients, and vice versa. While it appears reasonable to screen CD patients for AED at diagnosis, the latter may also develop after the start of GFD. Guidelines from the European Society for the Study of Celiac Disease suggest checking CD patients for thyroid disorders and T1DM at the diagnosis, and re-evaluate thyroid function every 24 months, also in patients without established thyroid disease. Other guidelines, however, do not provide such detailed recommendations [22]. Moreover, owing to the scarce and conflicting studies available, there is still no robust evidence on the preventive role of GFD on the later development of autoimmune disorders [23].

In the opposing scenario, repeated screenings for CD are strongly recommended for T1DM patients, but available data do not support such screening in patients with AIT [24].

Of interest, in 10 out of 13, T1DM was the first “driver” disease.

Presently, only retrospective studies would suggest a protective effect of an early start of GFD [25,26]. In particular, CD patients most at risk for autoimmune disease would be those diagnosed early in life and having a family history of autoimmunity, in whom GFD showed a protective effect [27].

However, a beneficial effect from GFD can be expected, as in general, both in children and adults, intestinal inflammation and a related dysbiosis are known to promote extraintestinal autoimmune diseases [28,29].

There is recent evidence that microbiota dysbiosis may be implicated in the autoimmunity induced by Foxp3+ regulatory T-cell deficiency and that microbiota remodeling significantly influences the outcome in autoimmune diseases [30,31].

Hence, any measure that can relieve intestinal inflammation in CD patients most likely will have a positive impact on the manifestation of glandular autoimmunity.

In a scenario in which CD screening is universally recommended for T1DM, but not for other AEDs, the diagnosis of CD relies on case-finding strategies. While the clinical presentation of CD in AED is classified as symptomless in approximately half of cases, a more accurate analysis often discloses a wide array of symptoms suggestive of CD [32,33].

In our series, about half of APS cases had iron-deficiency anemia, a frequent extraintestinal manifestation of CD. Of note, according to the 2021 British Society of Gastroenterology guidelines for the management of iron deficiency anemia in adults, the presence of this condition should be considered as a sign for the suspicion of CD and, thus, lead to appropriate testing [34].

In a sizeable proportion of cases, however, the diagnosis of CD was suspected based on non-classical manifestations of CD, such as recurrent miscarriages and low bone mass density. Both conditions can be detected by the same specialists that usually follow patients with AED and should trigger investigations for CD, as the prompt start of a GFD can rapidly reduce the risk of further miscarriages, and improve bone mineral density [35,36].

Importantly, the effects of the co-presence of CD might have a significant clinical impact since malabsorption associated with CD may lead to lability of diabetes and/or thyroid-related therapy efficacy, and thus complicate treatment [37,38].

Therefore, regardless of the first diagnosis, proper attention should be paid to the appearance of new signs or symptoms, or unsatisfactory responses to therapies, irrespective of the presence of classical gastrointestinal symptoms.

**Figure 1:**
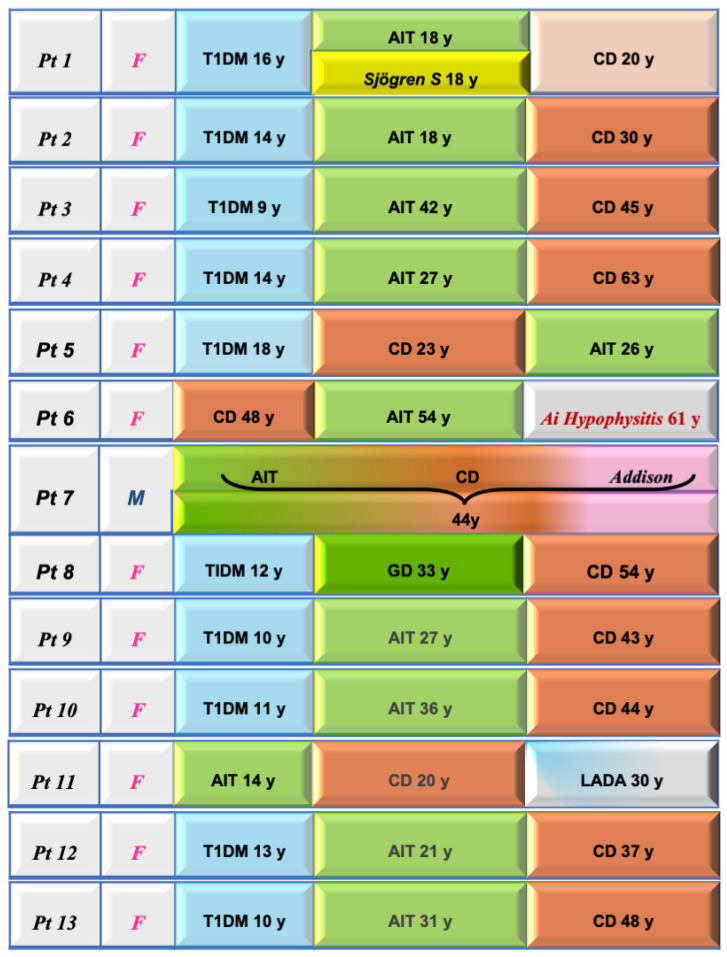
Clinical features and sequential diagnoses in the 13 celiac disease patients with associated autoimmune polyendocrine syndromes. In 10 out of 13, T1DM was the first “driver” disease. T1DM: type 1 diabetes mellitus; AIT: autoimmune thyroiditis; LADA: latent autoimmune diabetes of adults.

The detection of a monoglandular endocrinopathy in CD may only be part of an evolving and dynamic process with the appearance of other endocrinopathies at a later stage in CD as our study demonstrates (Figure 1).

Of clinical relevance, in the above-mentioned Italian study, 8% of APS patients developed cancers, breast and thyroid being the most common ones, proving that autoimmune diseases can be a condition conducive to the development of cancer [18,39].

A recent large cohort study highlighted the significant association between organ-specific immune-mediated diseases and risk of local cancer [40].

In conclusion, our study highlights the close association between CD and both monoglandular and polyglandular autoimmunity. In the era of non-invasive CD diagnosis, established for pediatric patients and proposed for adulthood [41,42,43], prospective studies to ascertain whether an early diagnosis of CD and a prompt GFD may positively impact the evolution and manifestation of glandular autoimmunity are mandatory.

## Figures and Tables

**Table 1:** Prevalence of autoimmune conditions in the study population (n = 920).

CONDITIONS	3
▶ Any autoimmune disease	299 (32.5%)
▶ Any autoimmune endocrinopathy	262 (28.5%)
✴ Autoimmune thyroiditis	223 (24.2%)
✴ Type 1 Diabetes Mellitus ✴ Latent Autoimmune Diabetes of the Adult	27 (2.9%) 2 (0.2%)
✴ Graves’ Disease	21 (2.3%)
✴ Addison’s Disease	1 (0.1%)
✴ Autoimmune Hypophysitis	1 (0.1%)
▶ At least two autoimmune endocrinopathies	13 (1.4%)

**Table 2:** Demographics of patients with associated autoimmune disease.

Autoimmune Disease (Total pts)	Female n (%)	Age at Diagnosis of CD Median (Range)
▶ Any autoimmune disease (n = 299)	254 (84.9%)	38.7 (16.1–76.8)
▶ Any autoimmune endocrinopathy (n = 262)	223 (85.1%)	38.0 (16.1–76.0)
▶ Autoimmune polyglandular syndrome (n = 13)	12 (92.3%)	43.8 (19.7–63.5)

Thirteen patients (1.4%) had two or more AEDs, qualifying for an APS diagnosis (Table 3 and Figure 1). Of them, 10 (77%) could be classified as APS type 3, 2 (15%) as APS type 4, and only 1 (7.7%) satisfied the criteria of APS type 2 having manifested the clinical onset of AD at 44 years old with concomitant diagnosis of AIT and celiac disease.

**Table 3:** Clinical and histological features of celiac disease in the 13 patients with associated autoimmune polyendocrine syndromes. T1DM: type 1 diabetes mellitus; AIT: autoimmune thyroiditis; LADA: latent autoimmune diabetes of adults.

Case	Sex	Age at the Diagnosis of CD (Years)	Reason Leading to CD Diagnosis	Other Manifestations of CD at the Diagnosis	Duodenal Histology (Marsh–Oberhuber)
1	F	20	Screening for T1DM patients	History of recurrent miscarriages	3b
2	F	30	Screening for T1DM patients	Low bone mass density	3a
3	F	45	Iron deficiency anaemia	History of recurrent miscarriages	3c
4	F	63	Iron deficiency anaemia, diarrhoea	Low bone mass density	1
5	F	23	Iron deficiency anaemia	Low bone mass density	3c
6	F	48	Weight loss, diarrhoea	None	3c
7	M	44	Screening for Addison’s disease	Recurrent oral apthosis	3c
8	F	54	Screening for T1DM patients	None	3b
9	F	43	Diarrhoea, iron deficiency anaemia	Low bone mass density	3b
10	F	44	Diarrhoea, weight loss, iron deficiency anaemia	Low bone mass density, history of recurrent miscarriages	3c
11	F	20	Iron deficiency anaemia	Low bone mass density	3c
12	F	37	Abdominal pain, iron deficiency anaemia	None	1
13	F	48	Abdominal pain, diarrhoea, skin rash	None	3b

**Table 4:** Prevalence of subtypes of adult form of autoimmune polyendocrine syndromes based on the combination of autoimmune endocrine disease [10,11]. APS: autoimmune polyendocrine syndrome. AIT: autoimmune thyroiditis. T1DM: type 1 diabetes mellitus.

APS Type (Defining Diseases)	N (% of APS)
▶ APS type 2 (Addison’s disease AND AIT)	1 (7.7%)
▶ APS type 3 (AIT AND T1DM)	10 (77%)
▶ APS type 4 (any combination of at least two AEDs not classifiable as other form of APS)	2 (15.4%)

## Data Availability

The data presented in this study are available on request from the corresponding author.
